# Bi_5_FeTi_3_O_15_ nanofibers/graphene nanocomposites as an effective counter electrode for dye-sensitized solar cells

**DOI:** 10.1186/s11671-016-1799-5

**Published:** 2017-01-06

**Authors:** H. W. Zheng, X. Liang, Y. H. Yu, K. Wang, X. A. Zhang, B. Q. Men, C. L. Diao, C. X. Peng, G. T. Yue

**Affiliations:** 1 School of Physics and Electronics, Institute of Microsystem, and Laboratory of Photovoltaic Materials of Henan Province, Henan University, Kaifeng, 475004 China; 2 Department of Electronic Information Engineering, Henan Vocational College of Agriculture, Zhengzhou, 451450 China

**Keywords:** Bi_5_FeTi_3_O_15_, Graphene, Nanocomposites, Counter electrode, Dye-sensitized solar cell

## Abstract

The present study reports Bi_5_FeTi_3_O_15_ (BFTO) nanofibers/graphene (Gr) nanocomposites (BGr) as counter electrodes (CEs) in dye-sensitized solar cells (DSSCs). BFTO nanofibers with diameters of 40–100 nm were fabricated by sol-gel based electrospinning technique. The microstructure and surface morphology of the BFTO nanofibers and the BGr nanocomposites were characterized by X-ray diffraction, scanning electron microscopy and transmission electron microscopy. The electrochemical performances of BGr CEs were comprehensively characterized and investigated. Compared to pristine BFTO, the nanocomposites have a marked improvement in electrocatalytic performance for the reduction of triiodide because of larger surface area and lower transfer resistance on the electrolyte-electrode interface. The maximum power conversion efficiency has reached 9.56%, which is much larger than that of pure BFTO CEs (0.22%).

## Background

In recent years, dye-sensitized solar cells (DSSCs) have attracted extensive attention as a potential alternative to silicon solar cells due to its high efficiency and low cost [1]. As a rule, the DSSC consists of three parts: a dye-sensitized TiO_2_ photoanode, an electrolyte including iodide/triiodide (I_3_^─^/I^─^) redox couples, and a counter electrode (CE) with excellent catalytic ability [2]. In general, the CE plays an important role in collecting the electron from an external circuit to catalyze the reduction of triiodide (I_3_^─^) to iodide (I^─^) in a DSSC [3, 4]. Consequently, the ideal CE material needs to have a high reduction catalytic activity, good chemical stability, low sheet resistance, and low production cost [5, 6]. Platinum (Pt) has been widely used as an ideal material for CE, however, the expensive price and limited reserves in nature have been the major concern for the energy community. Therefore, exploring replace Pt-based CE material in DSSC has attracted the attention of research institutions [7, 8].

Graphene (Gr) and its hybrids, which rely on their high thermal conductivity, excellent mobility of charge carriers, and extremely high theoretical specific surface area, have been widely used as CEs in DSSCs [9–11]. Recently, some materials including carbon nanotubes, poly(3,4-ethylenedioxythiophene), ZnO, TiO_2_, and NiCo_2_O_4_ composited with graphene have shown improved electrochemical behavior and power conversion efficiency compared to those without graphene [12–16]. But quaternary metal oxide Bi_5_FeTi_3_O_15_/grapheme composites have been rarely mentioned in previous literatures for DSSCs.

Bi_5_FeTi_3_O_15_ (BFTO) is a member of perovskite family, which exhibits a variety of interesting physical properties containing magnetic, ferroelectric, and dielectric properties [17–19]. BFTO is a kind of material with a direct bandgap (2.13 eV), high chemical stability and non-toxicity [20–22]. BFTO can be gained by inserting BiFeO_3_ into three-layered Bi_4_Ti_3_O_12_, forming a four-layered perovskite structure [23]. Bi_4_Ti_3_O_12_ and BiFeO_3_ have been found to be good candidates for DSSC [24, 25]. As a result, BFTO could also be utilized to build DSSCs. However, these ferroelectric oxides as CE in DSSC have scarcely been reported. In this work, we demonstrated BFTO/Gr nanocomposites as CE in DSSCs due to the low charge mobility and inferior catalytic activity of BFTO CE, expecting that Gr could promote the catalytic activity of the nanocomposites CE and thus enhance the photovoltaic property of the DSSCs based on the BFTO. This work could widen the potential applications of multibasic oxides in the photophysics and photochemistry field.

## Methods

All chemicals except graphene are of analytical grade. High purity (99.5%) graphene was purchased from Aladdin Industrial Corporation. Firstly, ethanol, bismuth nitrate, and iron acetylacetonate were dissolved in N, N-dimethylformamide. Then, tetrabutyl titanate and polyvinylpyrrolidone were slowly added in the solution stirring for 12 h to form the yellow precursor solution for electrospinning. The electrospinning parameters were setted as accelerating voltage of 10 kV and collecting distance of 20 cm, and the as-collected nanofibers were calcined in muffle furnace at 650 °C.

The TiO_2_, used as photoanode, was soaking through N719 dye-sensitized solution and dried in air. BFTO or BFTO/graphene (BGr) powder (0.1 g) was mixed in appropriate amount of PEG 2000 and ground constantly. Then, absolute ethanol (1 ml) was added and stirred to form a colloid. The CEs were gained by coating on FTO glass with the colloid followed by heating at 400 °C for 30 min under the protection of argon. BGr CEs were prepared with Gr of 0, 0.5, 1, 1.5 and 2 wt%, (labeled as BGr0, BGr0.5, BGr1, BGr1.5, BGr2), respectively. The effective areas of all cells were 0.25 cm^2^. Finally, the DSSC was assembled with prepared TiO_2_ photoanode like sandwich structure and was injected the electrolyte into the internal space between CE and photoanode.

A diffractometer with a Cu-Kα (with λ = 0.1542 nm) radiation source was used as the record of X-ray diffraction (XRD DX-2700) pattern for structures characterizing. The surface morphologies and microstructures were observed by using a scanning electron microscope (SEM, JSM-7001 F) and transmission electron microscope (TEM, JEM-2100). The electronic absorption spectra were recorded by a UV-vis photospectrometer (Varian Cary 5000). The photocurrent-voltage (*J*-*V*) curves of the DSSCs were recorded by a solar simulator under AM 1.5 illumination. The *J*-*V* curves, electrochemical impendence spectroscopy (EIS) and Tafel polarization curves were characterized by the electrochemical workstation (Wuhan CorrTest Instrument Co., Ltd.). Monochromatic incident photo-to-current conversion efficiency (IPCE) curves of devices were measured on an IPCE measurement system (Qtest Station 500ADX). The fill factor (FF) and the photoelectric conversion efficiency (*η*) of DSSC are calculated according to the following equations:

1 $$ \eta \left(\%\right)=\frac{J_{\max}\times {V}_{\max }}{P_{\mathrm{in}}}=\frac{J_{\mathrm{sc}}\times {V}_{\mathrm{oc}}\times \mathrm{F}\mathrm{F}}{P_{\mathrm{in}}}\times 100\% $$

2 $$ \mathrm{F}\mathrm{F}=\frac{J_{\max}\times {V}_{\max }}{J_{\mathrm{sc}}\times {V}_{\mathrm{oc}}} $$

Where *J*_sc_ is the short-circuit current density (mA cm^−2^), *V*_oc_ is the open-circuit voltage (V), *P*_in_ is the incident light power (mW cm^−2^), and *J*_max_ (mA cm^−2^), and *V*_max_ (V) is the current density and voltage at the point of the maximum power output in the *J-V* curves, respectively.

## Results and discussion

**Fig. 1:**
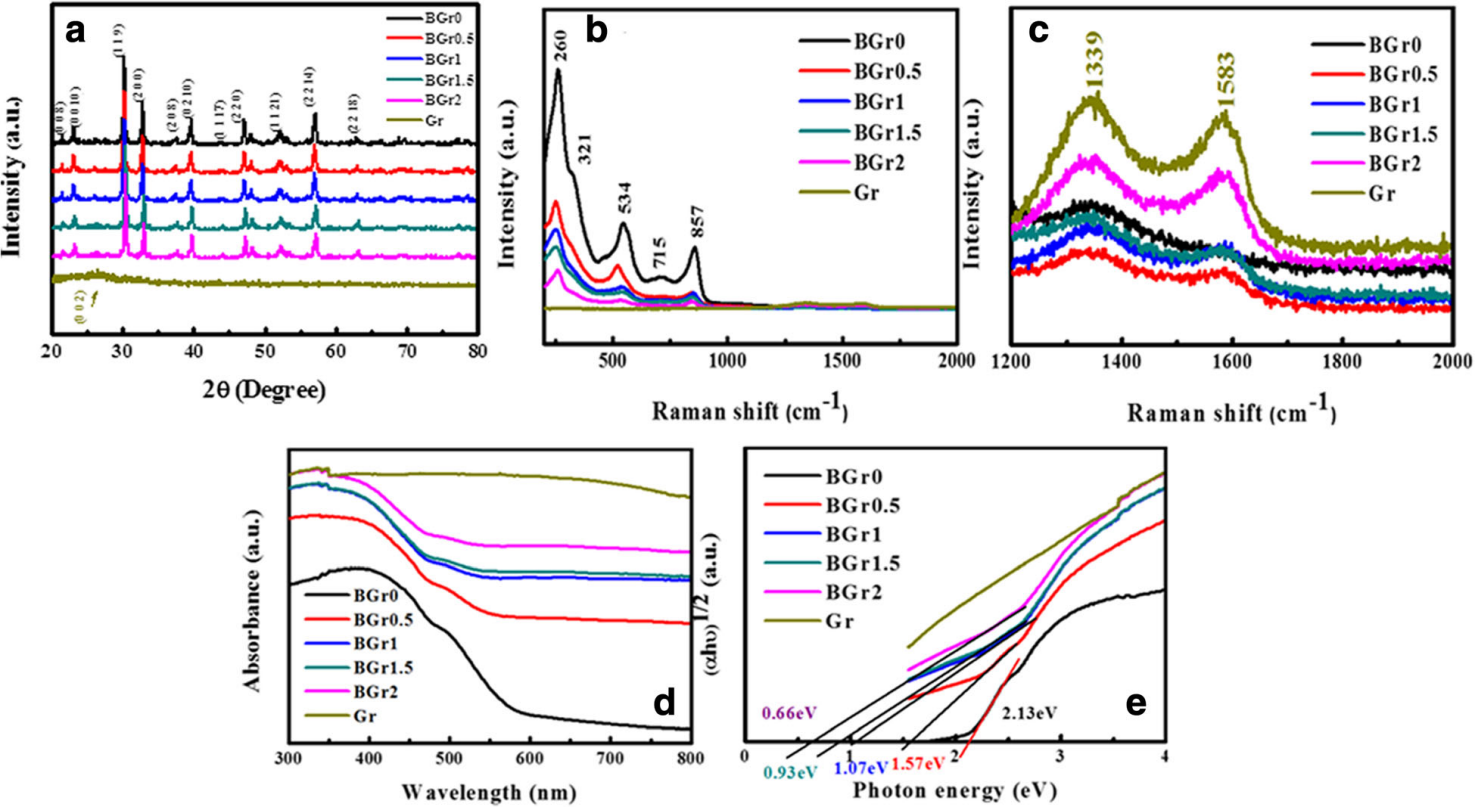
**a** XRD patterns of the BFTO, BGr, and Gr. **b** Room-temperature Raman spectra of BFTO, BGr, and Gr. **c** Partial enlargement drawing from (**b**). **d** UV-vis spectrum of BFTO, BGr, and Gr. **e** The plot of (*αhv*)^1/2^ as a function of photon energy *hv* around the absorption edge for BFTO, BGr, and Gr, respectively

Figure 1a presents the XRD patterns of pure BFTO nanofibers, Gr powders, and BGr nanocomposites, respectively. The positions and relative intensities of diffraction peaks are corresponding to JCPDS card NO.38-1257 and 65-6212, which indicate that the BFTO and Gr are pure phase within the limitation of XRD diffractometer, respectively. It is noted that the XRD patterns of BGr0.5, BGr1, BGr1.5, and BGr2 are almost the same and the peaks of Gr are not observed due to its less content. Raman spectra are shown in Fig. 1b and c to further verify the phase composition of the samples. For BFTO, the 260, 321, 534, and 857 cm^−1^ modes are resulted from the torsional bending and the stretching vibration modes of TiO_6_ octahedral. The origin of 715 cm^−1^ mode should be correlated to the Bi-Fe-O perovskite block because this mode has not been found in some Bi-layered oxides without Fe element such as Bi_4_Ti_3_O_12_ and CaBi_4_Ti_4_O_15_ [26, 27]. Moreover, the mode at 321 cm^−1^ could correspond to ferroelectric phase transition as reported previously [28]. From the Raman measurement result, it is reasonable to conclude that BFTO single phase with four-layered perovskite structure has been successfully prepared. As seen from Fig. 1c, there are two prominent peaks of Gr at 1339 and 1583 cm^−1^, which are assigned to the disordered (D) and graphitic (G) bands, respectively. The D peak is due to edge planes and disordered structure defect of lattice, while G peak belongs to the *E*_2g_ phonon of sp^2^ bonded carbon atoms [29, 30]. Consequently, the BFTO and Gr are successfully composited from the above Raman spectrum. Although 2D band is characteristic peak for graphene in Raman spectrum, in some literatures for graphene composited with inorganics, no 2D band was observed [31–33]. Moreover, in the references 15 and 31, the Raman measurement of graphene shows that the intensity of D-band (*I*_D_) is relatively higher than that of G-band (*I*_G_), while *I*_G_ is larger than that of *I*_D_ for the graphene oxide [34].

Figure 1d shows the UV-vis spectra of the BFTO, BGr, and Gr. According to the spectrum of BFTO, it could find that BFTO absorbs light from UV light to visible light shorter than 600 nm, which is consistent with its yellow appearance. According to the UV-vis spectra, the Gr has a perfect photoabsorption property in the whole visible light region. Therefore, BFTO compounded with Gr could display a better light-harvesting capability over the visible to near-IR region, which is also ascertained by the UV-vis spectra of BGr nanocomposites. The (*αhv*)^1/2^ versus *hv* plots are shown in Fig. 1e. By means of linear extrapolation method, the optical bandgap of BFTO nanofibers can be approximately estimated as 2.13 eV, which is comparable with that published in the previous literature. Moreover, it can be seen that optical bandgap of BGr decreases with increasing Gr content, which indicates the photoabsorption ability and the numbers of photogenerated carriers can be increased through the combination of Gr.

**Fig. 2:**
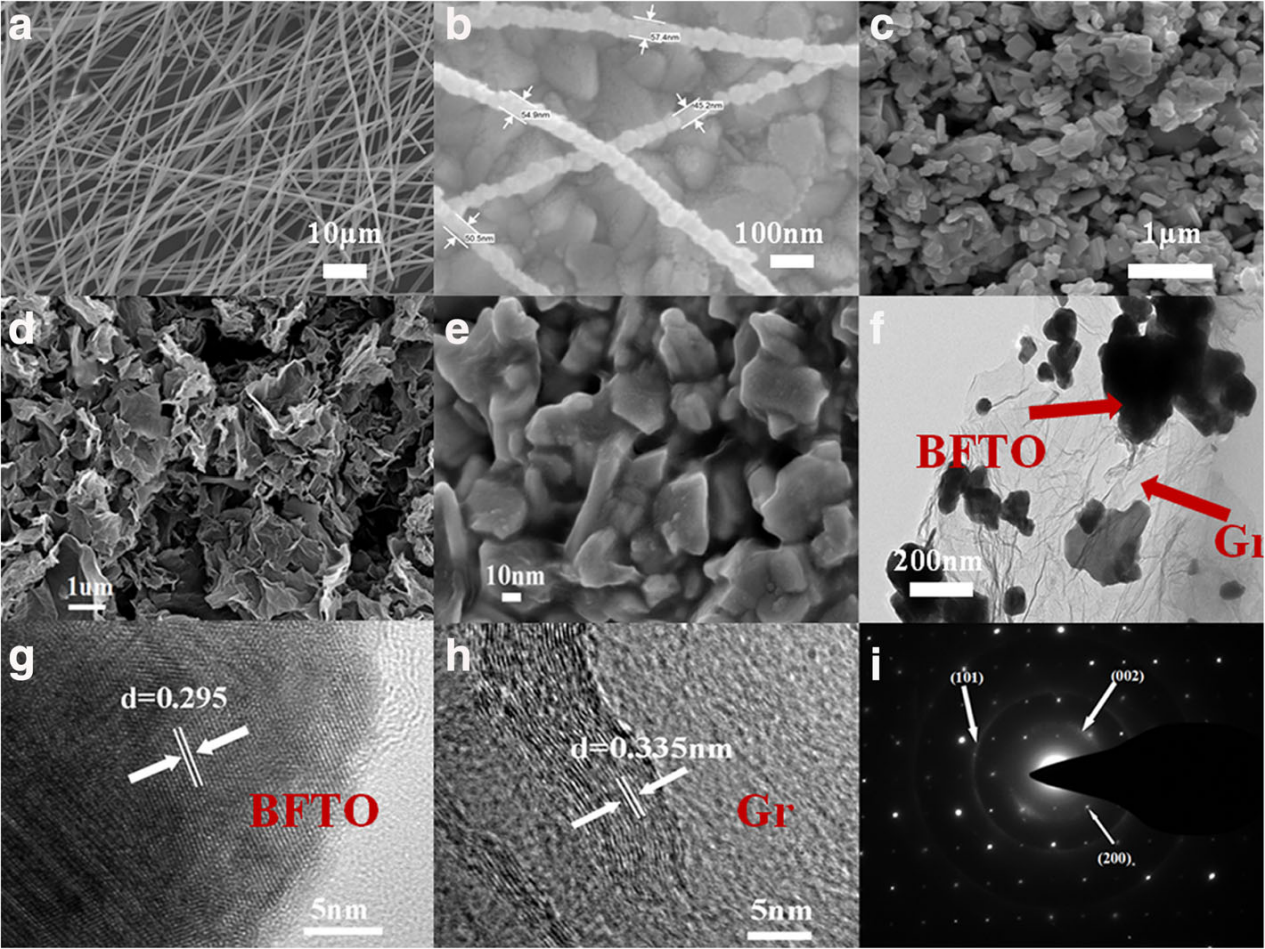
**a** Representative SEM images of unsintered nanofibers. **b** Gives the diameter of BFTO nanofibers after calculation. **c**–**e** SEM images of the BFTO, Gr, and typical BGr films as CEs. **f** Typical TEM micrograph of BGr. **g**, **h** HRTEM images of BFTO and Gr. **i** SAED patterns of BGr

The surface morphology and microstructure of BFTO nanofibers, BGr, and Gr are presented in Fig. 2. From Fig. 2a, the average diameter of the unsintered nanofibers is in the range of 100–300 nm, and their surface are smooth. After calcination, a continuous fine grained structure was observed and the average diameter of BFTO fibers is in the range of 40–100 nm. The SEM images of BFTO, Gr, and BGr films as CEs are displayed in Fig. 2c–e, respectively. It is obvious that the BFTO and Gr CEs are composed of nanoparticles and thin silk-like sheets. As shown in Fig. 2e, Gr sheets are commendably dispersed with the BFTO nanoparticles, which indicate the BGr nanocomposites have been successfully synthesized. The TEM image of typical BGr sample is exhibited in Fig. 2f, the black particles characterize the BFTO nanoparticles, and the reticulation denotes gray Gr. It can be inferred that BFTO and Gr are adequately mixed without change in crystalline structure. According to the high magnification TEM image in Fig. 2g, h, the lattice spacing is measured to be about 0.295 and 0.335 nm by the formula of *Rd* = *L*, matching with the (119) plane of orthorhombic BFTO and (002) reflection of Gr, respectively. This can be further confirmed by the selected area electron diffraction (SAED) image of typical BGr in Fig. 2i, which appears as the strong diffraction spots of BFTO and the diffraction rings of Gr.

**Fig. 3:**
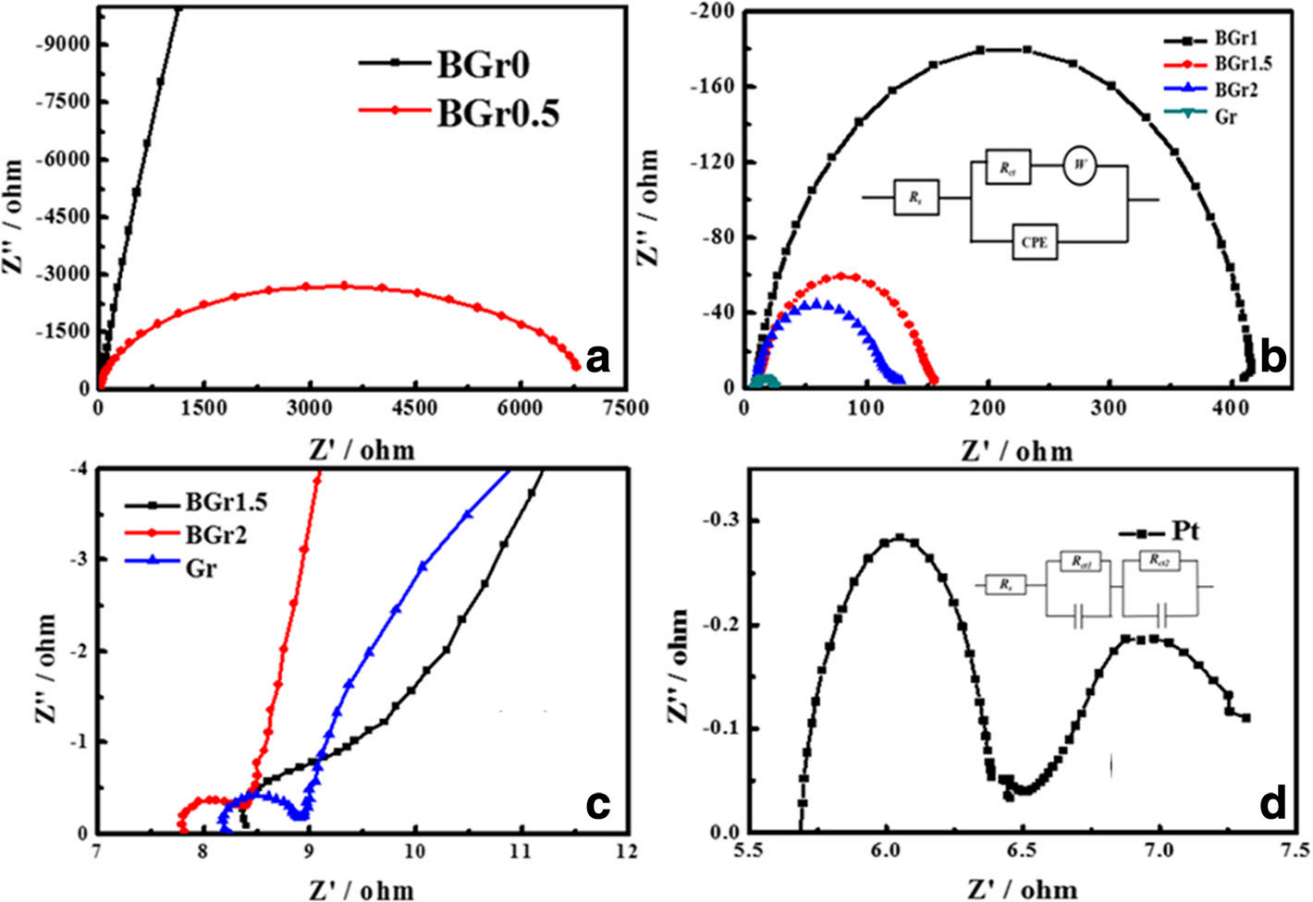
Nyquist plots (**a**–**d**) for symmetric cells fabricated with BGr0, BGr0.5, BGr1, BGr1.5, BGr2, Gr, and Pt CEs, and inset of (**b**) is equivalent circuit applied to fit the Nyquist plots

EIS measurement is performed in symmetrical cells fabricated with two identical CEs (CE/electrolyte/CE) to analyze the correlation between the electrocatalytic activity of the CE and quality of devices. Nyquist plots in Fig. 3a–c, and d clarify impedance characteristics of the DSSCs based on BGr0, BGr0.5, BGr1, BGr1.5, BGr2, Gr, and Pt CEs; and the corresponding electrochemical parameters are summarized in Table 1. The intercept on the horizontal axis stands for the series resistance (*R*_s_), a reflection of conductive substrate resistance, and lead resistance. The charge transfer resistance (*R*_ct_) at the CE/electrolyte interface is the intercept of the first semicircle, which characterizes the electrocatalytic ability of CEs for the reduction of triiodide [35], while the Nernst diffusion impedance corresponding to the diffusion resistance of the redox couples in the electrolyte is referred by the second arc. It is worthwhile to note that *R*_ct_ and *R*_s_ are the important parameters for appraising the performance of CEs. It is well known that a smaller *R*_s_ represents a higher conductivity and the smaller *R*_ct_, the lower ∆*E*_p_, bringing about a faster electron transfer from CE to electrolyte, thus the electrocatalytic activity can be enhanced. From Table 1, the *R*_s_ of BGr2 and BGr0 is 7.81 and 8.77 Ω cm^2^, respectively. Meanwhile, it is also observed that BGr0 and BGr0.5 have much larger *R*_ct_, implying that BGr0 and BGr0.5 have poor electrocatalytic ability. The *R*_ct_ of BGr2 is 0.82 Ω cm^2^, comparable with that of the reference Pt electrode (0.73 Ω cm^2^), indicating that BGr2 nanocomposite has an excellent electric conductivity and catalytic activity, which is an attribute to large specific surface area and high conductivity of BGr2 CE sample. Furthermore, the *R*_ct_ value for the BGr2 CE is much smaller than that of BGr0 CE, indicating that the former CE possesses more excellent catalytic activity and superior conductivity for I_*3*_^─^ reduction than pristine BFTO CE. In addition, it is clearly seen that the *R*_ct_ and *R*_s_ of the BTO/Gr CE are becoming smaller with increasing concentration of Gr, which presumably derives from the lamellar structure of graphene effectively promoting the electron transport and the diffusion of the redox electrolyte within the CEs, further resulting in better catalytic activity.

**Table 1:** Electrochemical parameters made from BGr0, BGr0.5, BGr1, BGr1.5, BGr2, Gr, and Pt CEs; and the photovoltaic properties of DSSCs based on the above CEs

CEs	*V*_oc_ (V)	*J*_sc_ (mA cm^−2^)	FF	*η*%	*R*_s_ (Ω)	*R*_ct_ (Ω)	IPCE (%)
BGr0	0.364	3.80	0.159	0.22	8.77	—	8.3
BGr0.5	0.770	8.51	0.386	2.53	8.70	6888.46	21.1
BGr1	0.720	15.79	0.437	4.97	8.43	400	39.4
BGr1.5	0.721	20.43	0.521	7.68	8.40	3.86	40.3
BGr2	0.740	21.56	0.599	9.56	7.81	0.82	42.3
Gr	0.744	22.78	0.663	11.23	8.25	0.74	44.8
Pt	0.780	24.02	0.562	12.21	7.06	0.73	48.4

**Fig. 4:**
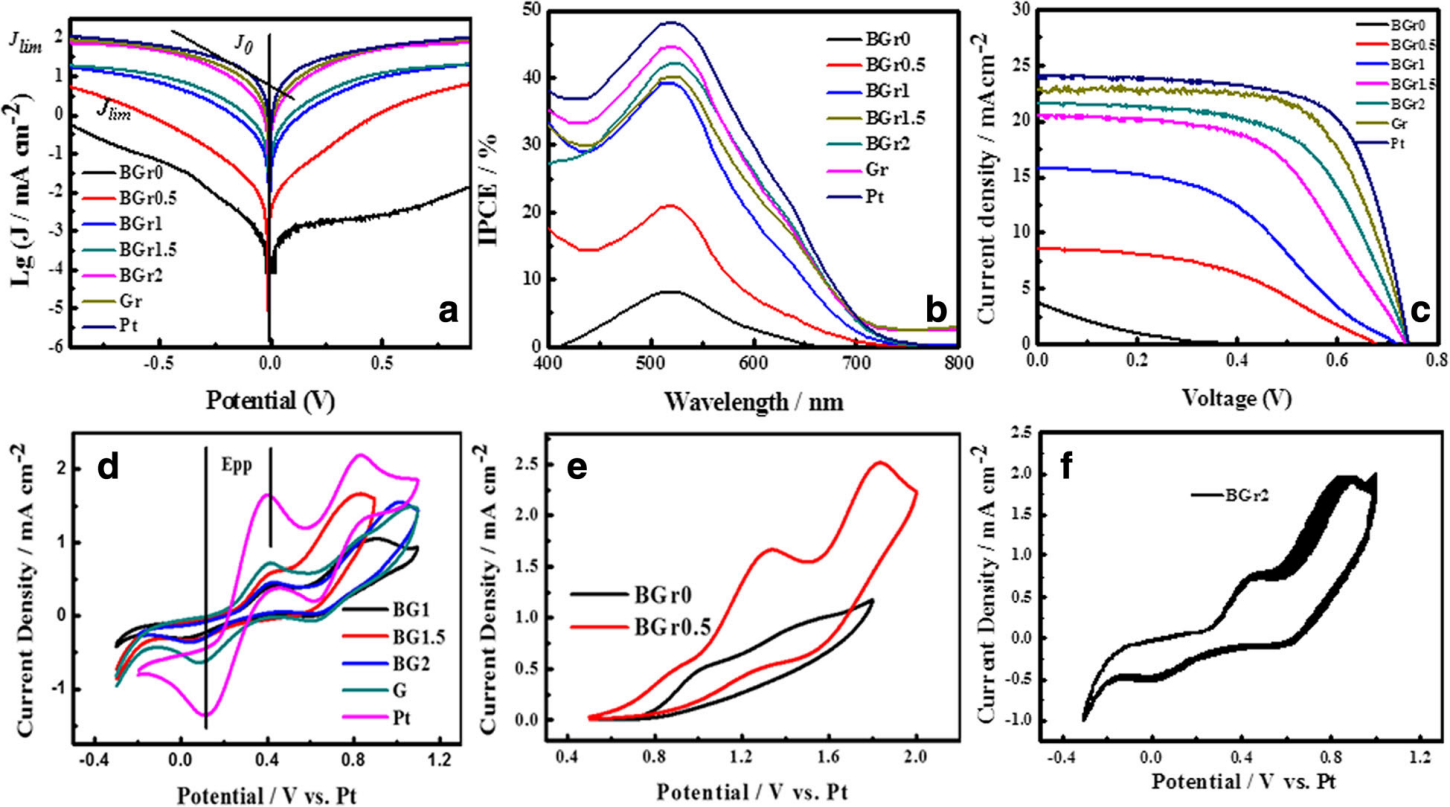
**a** Tafel-polarization curves of the symmetric cells fabricated with two identical CEs. **b** IPCE spectra of the DSSCs based on the BGr, Gr, and Pt CEs. **c** Current density-voltage curves (*J-V*). **d**, **e** CV curves for BGr, Gr, and Pt CEs. **f** Twenty cycles of CV curves from BGr2 CE at a scan rate of 50 mV/s

Tafel polarization curves are used to further investigate the catalytic activity of various CEs. As indicated in Fig. 4a, the exchange current density (*J*_0_) is approximately calculated by Tafel linear extrapolation method, namely, the intersection of the cathodic branch and the equilibrium potential line. The limiting current density (*J*_lim_) depends on the intersection of the cathodic branch and the vertical axis. The *J*_0_ and *J*_lim_ are closely related to the catalytic activity of catalysts, which can severally assess the reduction capability and the diffusion capability of the iodide/triiodide redox couple on CE materials [36]. Generally, a larger slope implies a higher *J*_0_. Obviously, the catalytic ability of various CEs is in the order of Pt > Gr > BGr2 > BGr1.5 > BGr1 > BGr0.5 > BGr0, suggesting that Gr is favorable for increasing the interfacial contact and decreasing the charge recombination rate by providing fast electron transport at CEs/electrolytes interfaces, thus enhancing the catalytic capability of pure BFTO CE for the reduction of triiodide, which is in consistent with the previous EIS results. Tafel polarization measurements further ascertain that Gr indeed improves the conductive ability and directly influences electrocatalytic activity of BFTO.

Figure 4b presents the IPCE of the DSSCs assembled with the assorted CEs. It can be seen that the strong photoelectric responses of DSSC with various CEs are all located at about 510 nm, and the photoelectric response enhances with the increasing of Gr content, which may probably due to the increased photogenerated carrier numbers originated from the increasing light absorption. The maximum photoelectrical responses of IPCE for the devices based on the BGr0 and BGr2 CEs are 8.3 and 42.3%, respectively. Since the values of IPCE are chiefly responsible by dye loading capacity and electron collection efficiency, it can be apparently distinguished that after Gr incorporation, more active sites are produced for the absorption of dye molecules and the photocurrent in the external circuit is enhanced, which increases the charge collection efficiency. As a result, the BFTO/Gr CEs have a faster electron transmission and a higher dye absorption capacity. Furthermore, the variation trend of the IPCE measurement is in a good consistent with the prior EIS results [37–39].

The *J-V* curves for the DSSCs fabricated with various CEs are shown in Fig. 4c, and the detailed photovoltaic parameters estimated from the *J*-*V* curves, including open-circuit voltage (*V*_oc_), short-circuit current density (*J*_sc_), fill factor (FF), and power conversion efficiency (PCE) are also summarized in Table 1. The photovoltaic parameters of DSSCs are gradually eased with increasing Gr content. When the mass fraction of Gr exceeds 2%, the PCE of DSSC remains nearly constant based on numerous photoelectric performance measurements. It is found that the DSSC assembled by BGr2 gives a PCE of 9.56%, *V*_oc_ of 740 mV, *J*_sc_ of 21.56 mA/cm^2^, and FF of 0.599, which is forty times greater than that of the DSSC based on the pristine BFTO CE (PCE = 0.22%, *V*_oc_ = 0.364 V, *J*_sc_ = 3.8 mA/cm^2^, and FF = 0.159). Consequently, Gr can advance the electrocatalytic activity of the BFTO CE which is in line with the former tests (EIS and Tafel curves). The improved photovoltaic performance of the DSSCs after Gr incorporation mainly ascribes to the following aspects: The contact frequency between the redox couple in electrolyte and the electrode can be accelerated owning to the large specific surface area of Gr, thus improve the electrolyte absorption ability and reaction speed. Then, the BGr CEs exhibit an improved electrocatalytic activity and electrolyte/electrode contact area compared with that of pure BFTO CE, leading to fast reaction kinetics and offering more electrocatalytic sites for the reduction of I_3_^−^ at the CE/electrolyte and a low charge recombination. Our study clearly demonstrates that small amount of Gr can significantly improve the electrochemical and photovoltaic properties of BFTO. Although the PCE value of BGr2 CE (9.56%) is smaller than that of Pt CE (12.21%), our work suggest that the incorporation of BFTO with Gr could be a promising and effective alternative to the noble Pt metal as a CE in DSSCs. Most recently, Gr are widely used to improve the photoelectrochemical properties of some metal oxides such as La_0.65_Sr_0.35_MnO_3_ and ZnO [40, 41], to our knowledge, it is the first time to describe Gr enhancing electrocatalytic activity and photovoltaic performance for four-component ferroelectric oxides.

Figure 4d, e shows the cyclic voltammetry (CV) curves of various BFTO based CEs, which is measured by using a three-electrode system with the Pt sheet as CE, saturated sliver chloride electrode as reference electrode, and various CEs as working electrode. For comparison, the CV curves for Pt and Gr are also displayed in Fig. 4d. Generally, a smaller overpotential (*E*_pp_) represents a better catalytic activity [42]. From Fig. 4d, e, the BGr2 CE has the lowest *E*_pp_ value among all BFTO-based CEs, reflecting that BGr2 CE has a decent catalytic activity. This is mainly due to Gr possesses a larger specific surface area which can hugely enhance the assessibility of the electrolyte to the electrode, thus improving interfacial charge transfer and enhancing the number of active catalytic sites, which is also complied with by the foregoing EIS, IPCE, and *J*-*V* measurement results. Moreover, 20 cycles of CV curves in Fig. 4f are employed to illustrate the stability of BG2 CE. It makes clear that BGr2 CE is fairly stable for catalyzing triiodide. The above experimental results imply that the incorporation of Gr can indeed improve the catalytic activity of BTO.

## Conclusions

In conclusion, Bi_5_FeTi_3_O_15_/graphene (BFTO/Gr) CEs have been successfully synthesized by a facile approach based on sol-gel and electrospinning techniques. The structure and morphology of the CEs were characterized by X-ray diffraction, scanning electron microscopy, and transmission electron microscopy. Extensive experiments indicate that BFTO/Gr CE has a vast enhancement of photovoltaic performance in comparison with pristine BFTO CE, which express as higher catalytic activity for the reduction of triiodide, larger specific surface area, and lower charge transfer resistance on the electrolyte-electrode interface, in which Gr plays a key role due to its inherent features. Under the optimum conditions, the largest PCE reaches 9.56% for the BGr2 CE in the DSSCs, which is 40 times larger than that of pure BFTO CE. This work provides a new, simple and effective means to improve the photoresponsivity of commonly four-component ferroelectric oxides, which may develop the application area of multifunctional metal oxides.

## Abbreviations

BFTO: Bi_5_FeTi_3_O_15_
BGr: BFTO nanofibers/Gr nanocomposites
DSSCs: Dye-sensitized solar cells
EIS: Electrochemical impendence spectroscopy
FF: Fill factor
Gr: Graphene
*I*_D_: The intensity of D-band
*I*_G_: The intensity of G-band
IPCE: Monochromatic incident photo-to-current conversion efficiency
*J-V*: Photocurrent-voltage
SEM: Scanning electron microscope
TEM: Transmission electron microscope
XRD: X-ray diffraction
*η*: Photo-electric conversion efficiency
